# Research progress on the association between Hashimoto’s thyroiditis and autoimmune encephalitis: a review

**DOI:** 10.3389/fimmu.2026.1896060

**Published:** 2026-09-09

**Authors:** Xin’ai Li, Jingwei Zhou, Jinxiang Fu, Junhui Wang, Zhiguo Ding

**Affiliations:** 1 Department of Thyropathy, Dongzhimen Hospital, Beijing University of Chinese Medicine, Beijing, China; 2 The 1st Ward, Department of Nephrology and Endocrinology, Dongzhimen Hospital, Beijing University of Chinese Medicine, Beijing, China; 3 Dong xiao kou Community Health Service Center, Beijing, China; 4 Thyropathy Hospital, Sun Simiao Hospital, Beijing University of Chinese Medicine, Shanxi, China; 5 Lunenfeld-Tanenbaum Research Institute, Mount Sinai Hospital, Toronto, ON, Canada

**Keywords:** autoimmune encephalitis (AE), diagnosis, Hashimoto’s encephalopathy, Hashimoto’s thyroiditis (HT), immune mechanism, treatment

## Abstract

Hashimoto’s thyroiditis (HT), which is the most common autoimmune thyroid disease, has, in recent times, been the subject of growing interest because of its possible link with autoimmune encephalitis (AE). This overview comprehensively reviews the epidemiological connections, shared immunopathological mechanisms, common clinical manifestations, diagnostic issues, and treatment methods of HT and AE. By combining the most recent progress in immunology, brain imaging, and clinical studies, we analyze the similarities and differences between HT-associated encephalopathy, commonly identified as Hashimoto’s encephalopathy, and the standard types of AE. Despite these advances, diagnostic and therapeutic challenges remain in patients who show neurological symptoms in the context of HT. The aim of this paper was to enhance the knowledge of clinicians about the connection between these two autoimmune disorders, normalize diagnostic procedures, and update treatment methods in order to achieve better patient results. Furthermore, this paper outlines some of the directions for future work that would help reveal mechanisms and develop clinical management.

## 1 Introduction

Hashimoto’s thyroiditis (HT), a common organ-specific autoimmune disorder, is mainly characterized by the presence of lymphocytes in the thyroid gland and increased levels of thyroid autoantibodies, mainly anti-thyroid peroxidase antibodies (TPOAbs) and anti-thyroglobulin antibodies (TgAbs) (1). Majority of the patients are hypothyroid at diagnosis. However, a small number of patients with HT develop neurological symptoms that are consistent with Hashimoto’s encephalopathy (HE), a steroid-responsive encephalopathy characterized by cognitive impairment, seizures, and psychiatric manifestations. The association between HT and autoimmune encephalitis (AE) has received more attention recently because both diseases may share overlapping immunopathogenic mechanisms including gene susceptibility, molecular mimicry, and blood–brain barrier (BBB) disruption (2, 3). Nevertheless, it remains a challenge to clinically separate HE from classical AE, such as anti-*N*-methyl-d-aspartate receptor (NMDAR) and anti-leucine-rich glioma-inactivated 1 (LGI1) encephalitis, particularly when neuronal surface antibodies (NSAbs) cannot be detected. The treatment of HE mainly relies on corticosteroids, whereas immune therapies such as intravenous immunoglobulin (IVIG), plasmapheresis, or rituximab are frequently used for AE (3). Therefore, a misdiagnosis or a late diagnosis of AE in patients with HT may result in ineffective treatment and more serious disease complications. This review summarizes the literature on the epidemiology, immunopathogenesis, and clinical features of HT-associated encephalitis, with a special focus on differential diagnosis and therapeutic approaches to increase clinical recognition and management.

For the literature search strategy, a comprehensive literature search was conducted using the PubMed, Web of Science, and Scopus databases, covering publications from January 2000 to December 2025. The search terms included combinations of “Hashimoto’s thyroiditis,” “autoimmune encephalitis,” “Hashimoto’s encephalopathy,” “steroid-responsive encephalopathy associated with autoimmune thyroiditis (SREAT),” “immune mechanisms,” “diagnosis,” and “treatment.” The reference lists of key articles were also manually searched to identify additional relevant studies. Due to the rarity of HE/SREAT, case reports and small case series were included alongside cohort studies, systematic reviews, and consensus guidelines. However, this limitation should be considered when interpreting the evidence presented in this review.

## 2 Epidemiological association between HT and AE

### 2.1 Comorbidity rate and population distribution characteristics

Epidemiological studies have consistently shown a strong correlation between HT and AE. This association suggests a potential epidemiological link between HT and AE, although a direct causal relationship has not been established. Thorough neurological assessments in patients with HT presenting with neurological symptoms are warranted. HE or SREAT is an extremely rare but, at the same time, a significantly clinically recognizable neurological complication of HT. In individuals with AE, thyroid autoantibody seropositivity has been recorded at levels higher than those observed in the general population (4). The symptoms of HE/SREAT are notably diverse and include neurological and neuropsychiatric manifestations: acute/subacute encephalopathy, cognitive impairment, seizures, mood changes, and movement disorders such as cerebellar ataxia or extrapyramidal symptoms (5). HE is extremely difficult to diagnose because it is rare, it can present in several different ways, and other causes of encephalopathy (e.g., infectious, metabolic, and other autoimmune causes) have to be ruled out first. The presence of high serum concentrations of TPOAb and TgAb is the main diagnostic criterion. At the same time, their pathological role is far from being clear (1, 6, 7). It should be noted that these antibodies have not only been found in serum but, more rarely, also in the cerebrospinal fluid (CSF), raising the possibility of their production in the brain or the disruption of the BBB. The higher incidence in women and the age pattern among patients with HE are consistent with the features of HT and with their shared autoimmune nature (8). Moreover, the high percentage of thyroid autoantibody positivity in patients with AE may suggest a predisposition toward co-occurrence, which could be dually explained by common immunogenetic predispositions or environmental exposures. Besides steroids, other immunosuppressive therapies such as IVIGs or plasmapheresis need to be considered in the treatment of patients with HE, in particular in those who do not respond to steroids (1). The discovery of HE as an identifiable clinical syndrome related to HT makes the case for clinicians to include autoimmune thyroiditis in the differential diagnosis of unexplained encephalopathy, particularly if it occurs in middle-aged women together with neuropsychiatric symptoms and increased thyroid autoantibodies. The coincidence of HT and AE in terms of comorbidity and demographics, especially the overlap of HE, supports HT as a possible risk factor for AE (9, 10). This underlines the importance of a neurological workup and testing for thyroid autoantibodies in cases of unexplained encephalopathy for early diagnosis and treatment with immunotherapy. Clarifying the immune pathogenesis and determining biomarkers for risk assessment and treatment guidance are areas for further research (1, 11–14). It should be acknowledged that much of the epidemiological evidence derives from case reports and small case series, which limits the strength of these epidemiological associations.

### 2.2 Overlapping genetic susceptibility

The genetic overlap in susceptibility to certain autoimmune disorders suggests that there are common immunogenetic elements underlying the pathogenesis of both disorders. The focus of this genetic intersection is the human leukocyte antigen (HLA) gene complex, in particular its class II region. This area contains highly polymorphic genes that encode molecules essential for antigen processing and presentation to T helper (CD4^+^) effector cells, establishing it as the strongest genetic susceptibility locus for many autoimmune diseases including HT and AE. The specific HLA alleles that mediate these effects include HLA-DR3 and HLA-DR4, whose associations are well established in HT. However, their role in specific AE subtypes has been reported in only a limited number of studies and requires further investigation. The identification of these alleles in different ethnic and racial groups worldwide, as well as their consistent association with HT, with emerging reports in certain AE subtypes, reflects their potential role in disease susceptibility. It should be emphasized that not all forms of AE share the same genetic background and that genetic predisposition may vary across different AE subtypes. Through binding to distinct antigenic peptides, the HLA molecules dictate T-cell priming and tolerance failure. In addition, the preferential use of certain T-cell receptor β-chains by expanded autoreactive T cells in the central nervous system (CNS) of patients with AE indicates that specific T-cell clonotypes may be involved in the pathogenesis (15). These common allelic associations implicate these haplotypes in the predisposition to a variety of autoimmune diseases by allowing for abnormal immune recognition of self-antigens.

Apart from HLA, several immune gene loci have been identified by genome-wide association studies (GWAS) as common to both HT and AE. This fact further confirms the hypothesis that both diseases have a similar genetic background in the immune system. Points of interest are polymorphisms in the genes *CTLA-4*, *PTPN22*, and *IL-2RA*, as their mutations or variants have been linked to an increased risk of developing both HT and AE. The immune inhibitory receptor CTLA-4 plays a major role in dampening T-cell responses and maintaining self-tolerance, and its associations have been well documented in HT, while reports in AE remain limited to isolated case reports. More recently, the case of a young girl with a homozygous *CTLA-4* mutation presenting with isolated α-amino-3-hydroxy-5-methyl-4-isoxazolepropionic acid (AMPA) receptor encephalitis has been reported (16). The protein tyrosine phosphatase encoded by *PTPN22* negatively regulates T-cell receptor signaling, whereas C1858T polymorphism in *PTPN22* is an established susceptibility marker for HT, as evidenced by an animal model of human diseases involving trisomy 21 where this variant was associated with hypothyroidism and positive antibodies (17). However, its association with specific AE subtypes has been less extensively studied. The *IL-2RA* gene coding for interleukin-2 receptor alpha chain is a vital component for the function of regulatory T cells (Tregs) and overall immune tolerance, and its polymorphisms have been linked to autoimmune thyroid diseases and AE. These common genetic loci emphasize T-cell activation pathways and immune tolerance defects as the key mechanisms underlying both HT and AE. Apart from the genetic overlap, familial aggregation provides additional proof for a link between HT and AE. Family members of patients with HT have a greater likelihood of AE, which indicates that genetic factors inherited from parents play a role in the concurrence of these two autoimmune conditions. Despite AE being a rare disorder overall, the increased risk of HT in family members sheds light on the existence of shared susceptibility loci that predispose to autoimmunity in both the thyroid and the CNS. This is in line with the multi-hit model of autoimmunity where environmental factors trigger genetically predisposed individuals to initiate the disease process (18). The identification of specific HLA haplotypes and immune regulatory gene variants in familial clusters highlights the need for genetic screening and counseling in families that are affected by HT and AE. In addition to the genetic changes themselves, HT and AE also share similar features in the function and expression of the immune system. Both diseases feature the inappropriate activation of T lymphocytes and the generation of autoantibodies that target tissue-specific antigens. For example, autoantibodies in AE may target synaptic proteins as NMDA or AMPA receptor, leading to neuropsychiatric symptoms, whereas the antibodies in HT predominantly target thyroid peroxidase and thyroglobulin antigens (19). The genetic predisposition to dysregulated T-cell activation and defective immune tolerance underpinned by haplotypes and variants of the HLA, as well as other implicated genes, contribute to the loss of self-tolerance in the affected tissues. Moreover, overlapping genetic risk factors enable the use of shared therapeutic interventions. It has recently been shown that IgG4-related autoimmune diseases including AE respond well to B-cell depletion therapies, e.g., rituximab, thus indicating that targeting the common immunopathological pathways could be beneficial for patients with HT and AE (15). Knowledge of the genetic landscape of these diseases is a helpful tool for the customization of treatments and prognostication. Shared HLA-DR3/DR4 and non-HLA genes (e.g., *CTLA-4*, *PTPN22*, and *IL-2RA*) link HT and AE, with familial studies confirming genetic predisposition, highlighting shared mechanisms and therapeutic targets. These common genetic loci should be interpreted with caution as the associations vary across different AE subtypes and ethnic populations and the evidence is predominantly derived from individual case reports and small cohort studies.

### 2.3 Combined role of environmental triggering factors

Environmental factors are integral to the initiation or progression of HT and AE pathogenesis. Furthermore, they serve as triggers that can both initiate and exacerbate the autoimmune responses in these conditions. Among the various environmental factors, viral infections such as Epstein–Barr virus (EBV) and cytomegalovirus (CMV) are considered to play a key role as agents responsible for the induction of autoimmunity that leads to HT and AE. These viruses are capable of activating autoreactive T cells and B cells possibly by molecular mimicry, in which their antigens closely resemble self-antigens, which results in the immune system losing tolerance to self, leading to thyroid destruction and also to CNS autoimmunity. Numerous studies have linked EBV to autoimmune diseases, of which HT is just one example. The virus can increase B-cell activation and survival, thus possibly supporting the production of antibodies that target thyroid antigens. In the same way, AE cases showing anti-NMDA receptor encephalitis have been reported to be triggered by herpes simplex virus (HSV) infection. This condition strongly suggests that viral-induced immune activation may be the cause of CNS autoimmunity (20, 21). The molecular mimicry model has been substantiated by experimental data derived from models exposed to repeated infections, showing that Th17 cells become migratory to the CNS, subsequently leading to BBB dysfunction and an ability of pathogenic autoantibodies for entry (22).

Physiological stressors such as pregnancy and the postpartum period have been proven to cause immune alterations that can trigger or worsen HT and AE. Immunologically, pregnancy is characterized by the immune tolerance mechanisms that mainly help in the acceptance of the semi-allogeneic fetus while at the same time is associated with a shift in the immune response towards anti-inflammatory pathways. Postpartum immune reconstitution and rebound pro-inflammatory phenomena both appear to be responsible for inducing autoimmune disease exacerbations in the postpartum period. The predominance of postpartum HE occurrence among rare AE cases associated with HT emphasizes the contribution of hormonal and immune cell changes in disease development (23). It has also been demonstrated that stress, whether psychological or physical, can trigger immune changes, leading to loss of immune equilibrium and to increased autoreactive lymphocyte activation and cytokine production, resulting in the promotion of the autoimmune processes observed in both HT and AE. Both iodine excess and selenium deficiency have long been recognized as environmental risk factors for the development of HT. Excessive iodine intake can stimulate thyroid antigenicity, thereby encouraging lymphocytic infiltration, whereas selenium deficiency compromises the antioxidant defenses, thus increasing oxidative stress and inflammation in the thyroid gland. Micronutrient imbalances also affect CNS autoimmunity through oxidative stress and inflammatory pathways. In fact, oxidative stress has been implicated in BBB breakdown and neuroinflammation in AE; thus, environmental factors affecting redox balance could influence disease susceptibility and progression (24). Additional studies are needed to determine the exact mechanisms by which iodine and selenium status are able to modulate AE disease susceptibility. To summarize, EBV, CMV, pregnancy, iodine, and selenium induce HT and AE through immune dysregulation and therefore represent potential intervention targets in susceptible individuals.

## 3 Immunopathological mechanisms of HT and AE

### 3.1 Molecular mimicry and cross-reactivity

Molecular mimicry between thyroid antigens and CNS proteins has been hypothesized as a potential mechanism through which HT might contribute to the development of AE, which is generally known as HE or SREAT. The main autoantigen in HT, i.e., thyroid peroxidase (TPO), has sections of its amino acid sequence that are similar to myelin basic protein (MBP), which is the major component of CNS myelin sheaths. Such similarity could promote the activation of T cells that react against one’s own cells and the production of antibodies that are capable of cross-reacting with thyroid and neuronal tissues.

Anti-TPO antibodies, which are most often present in the blood in increased amounts in HT, have been found to be able to attach to astrocytes and neurons in the brain. They also have the potential to trigger complement activation and antibody-dependent cell-mediated cytotoxicity (ADCC) (14). A few cases of these antibodies being found in the CSF have been reported, which indicates the presence of CNS involvement in the manifestation of symptoms such as seizures, cognitive decline, and neuropsychiatric symptoms (14). Bioinformatics studies have discovered many proteins that are expressed in the CNS and which have sequence homology with thyroid autoantigens. Such proteins may explain the wide range of clinical phenotypes of HE/SREAT (7). That there are only very few cases of HE despite the presence of thyroid autoantibodies in a large number of people could be explained by the fact that these antibodies have to pass through the BBB in quite large amounts in order to result in CNS pathology. To summarize, the mimicry at the molecular level between thyroid autoantigens and CNS proteins helps in the formation of cross-reactive antibodies that are able to cross the BBB and lead to neuroinflammation via complement activation and ADCC. This scenario is at the base of the various clinical symptoms of HE and provides the rationale for immunosuppressive therapies. However, it is important to note that much of the evidence for molecular mimicry comes from bioinformatics sequence analyses and limited experimental studies, and direct clinical evidence demonstrating pathogenic cross-reactivity remains to be established.

### 3.2 T-cell-mediated immune dysregulation

T-cell-mediated immune dysregulation is a key factor for the onset of HT and may also be implicated in the development of AE, thus showcasing a common immunopathogenic feature of these conditions. Tregs play a vital role in preventing the immune system from attacking the body’s own cells. When these cells are deficient in HT, self-reactive T cells may escape peripheral tolerance. These cells could potentially migrate from the thyroid gland to the CNS, where they contribute to the neuroinflammation observed in AE (25, 26). Th17 cells, a lymphocyte subset that produces interleukin-17 (IL-17), represent another important player in the pathophysiology of both HT and AE. Studies have revealed an increase in Th17 cells and IL-17 levels in the blood of patients with HT and in the brains of patients with AE, these findings being associated with the promotion of neuroinflammation through BBB breakdown and increased influx of immune cells into the brain parenchyma (22, 25). Cytotoxic T lymphocytes (CTLs), in particular CD8^+^ T lymphocytes, are among the immune cells responsible for tissue damage in both HT and AE. The presence of these cells in the thyroid tissue of HT patients and the brain tissue of AE patients, especially in those whose disease is associated with intracellular antigen targets, implicates a shared cytotoxic mechanism supporting the immunopathology (27, 28). To summarize, disturbances in the T-cell subsets, such as reduced tolerance due to the lack of Tregs, increased production of pro-inflammatory Th17 cells, and infiltration of CD8^+^ T cells, are the main immunopathogenic pathways that bridge HT with AE. In-depth comprehension of these common mechanisms may open doors to new therapeutic approaches for patients suffering from both diseases.

### 3.3 Blood–brain barrier dysfunction

HT is a systemic autoimmune inflammatory disease that not only results in thyroid damage but also affects the CNS through BBB structural changes. Patients with HT have increased levels of pro-inflammatory cytokines in their blood, such as tumor necrosis factor-alpha (TNF-α) and interleukin-6 (IL-6), which can induce changes in the expression of the adhesion molecules (e.g., ICAM-1 and VCAM-1) on the surface of the endothelium in the brain vasculature, thus enabling immune cells to cross over and cause further damage, resulting in increased BBB permeability. Due to this greater permeability, thyroid-specific autoantibodies and activated lymphocytes enter the CNS and therefore lead to triggering or increasing the severity of AE (29). Autoantibodies targeting alpha-enolase (ENO1Ab) play a role in BBB damage via decreasing the levels of key tight junction proteins (e.g., claudins and occludins) in endothelial cells, thus compromising the BBB barrier function and allowing entry of neurotoxic substances into the brain (29). Imbalances in the thyroid hormones, which are commonly manifested in HT, such as in subclinical hypothyroidism, are also capable of impairing the BBB integrity by downregulating the expression of tight junction proteins (29). Neuroimaging is one of the most important investigations in clinical practice that indirectly captures BBB disruption. Gadolinium contrast enhancement on magnetic resonance imaging (MRI) adequately reflects sites of BBB breakdown, which also correlate with clinical symptoms on neurological examination (29). Diffusion tensor imaging (DTI) revealed brain regions with an altered microstructure that are involved in working memory and motor skills (30). In conclusion, the dysfunction of BBB in HT is a combination of cytokines, thyroid hormone derangements, and autoantibody-mediated injury resulting in increased permeability, allowing immune elements into the CNS. Imaging confirms the importance of BBB integrity in the neurological manifestations of HT. Gaining further insights is essential for the development of treatment strategies to safeguard the BBB and prevent CNS involvement. It should be noted that the evidence for BBB disruption in HT is partly indirect and is derived from neuroimaging studies and animal models. The exact contribution of BBB dysfunction to the pathogenesis of HE requires further investigation.

### 3.4 Potential role of neuronal surface antibodies

**Figure 1:**
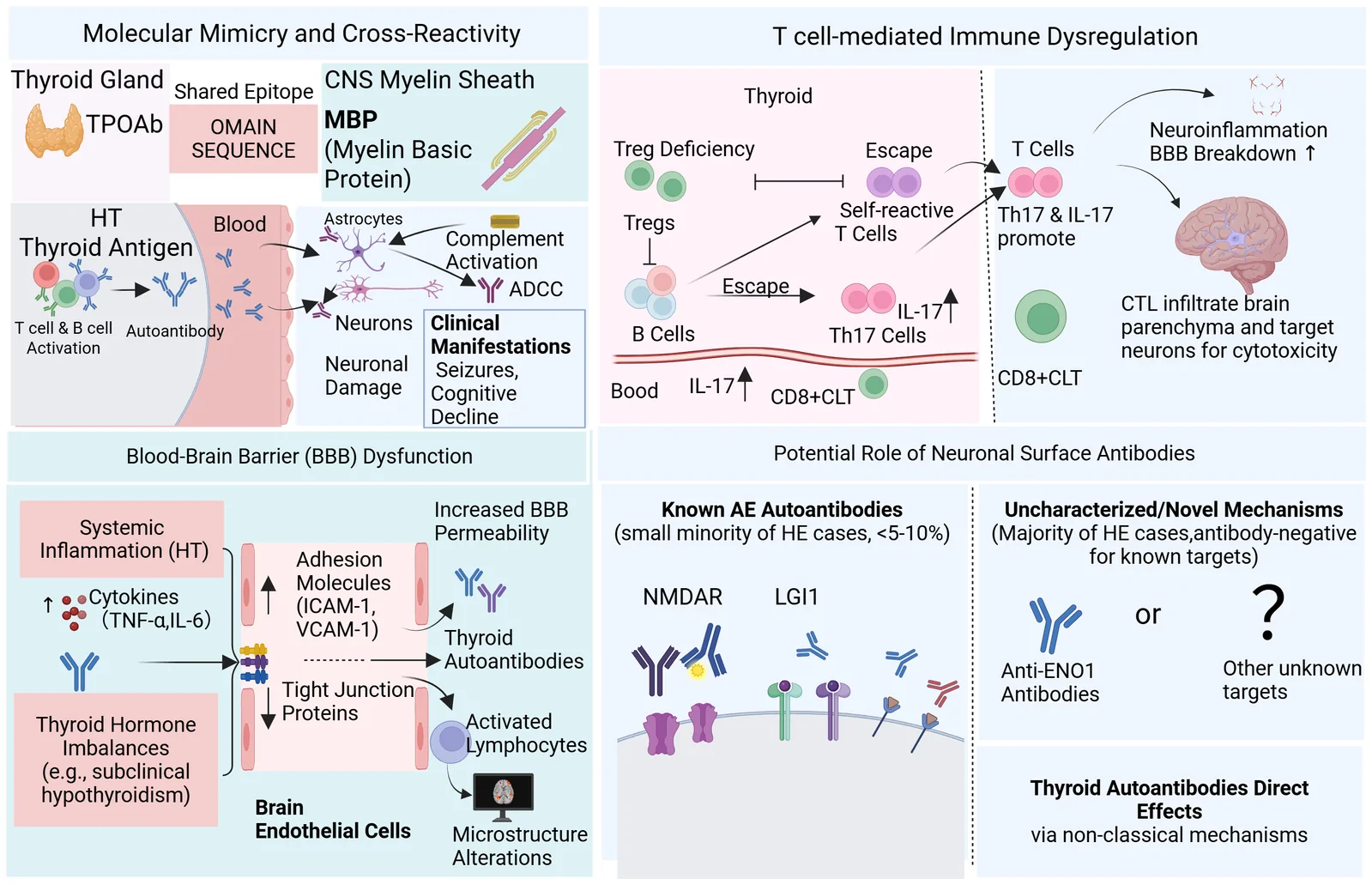
Pathophysiological mechanisms linking Hashimoto’s thyroiditis to autoimmune encephalopathy.

The discovery of NSAbs in certain HE cases offers a perspective into the immunopathogenic link between HT and autoimmune CNS disorders. Some patients with HE have antibody in the serum that reacts with the neuronal surface antigens that have been characterized, including NMDAR and LGI1, the main antibodies in AE (31–36). This could mean that HT acts as a factor triggering the production of neuronal autoantibodies or that both disorders share B-cell activation pathways. The fact is, the majority of patients with HE do not have detectable antibodies to the known neuronal surface antigen, suggesting either unknown antibodies or other pathogenic mechanisms. The antibody targeting α-enolase has been described in HT cases, which could represent a new target for autoimmune brain dysfunction. In addition, thyroid autoantibodies may directly have an effect on neuronal function through non-classical mechanisms. In summary, although NSAbs are present only in a minority of HE cases, the absence of known antibodies in the majority of cases suggests the involvement of novel autoantibodies or alternative pathogenic mechanisms. Further studies are needed to characterize these novel antibodies and to understand their functions. This, in turn, might pave the way to better diagnosis and treatment of patients with neurological manifestations associated with HT. Importantly, the pathogenic role of thyroid autoantibodies in HE remains unproven, and their presence may serve as a marker for autoimmune tendency rather than as direct mediators of neural injury (**Figure 1**).

## 4 Clinical features of HT-associated encephalitis (Hashimoto’s encephalopathy)

### 4.1 Clinical heterogeneity of HE

HE/SREAT is a rare autoimmune neurological disorder associated with HT and is highly heterogeneous clinically. This explains why it can show a very wide spectrum of clinical manifestations. Typical signs of HE may even be mistaken for other forms of AE as the patient may present with a rapidly developing or a subacute impairment of cognition, psychiatric disturbance, seizures, or various other movement disorders. Cognitive symptoms generally consist of memory and concentration problems, and the decline can be either rapid or slow. Psychiatric disorders such as depression, hallucinations, and psychosis may masquerade as primary psychiatric disorders. Seizures may be a focal or a generalized type and may turn into status epilepticus. Movement disorders including myoclonus, tremor, or ataxia, among others, frequently occur, and there may even be stroke-like episodes. Significantly, fewer faciobrachial dystonic seizures (FBDS) have been reported in HE compared with those in anti-LGI1 encephalitis, while cognitive and psychiatric symptoms appear to be more expressive. The wide heterogeneity of the disorder is evidenced by case series where the HE pattern was demonstrated between monophasic episodes and relapse. The pathophysiology of the disease is autoimmune inflammation. Autoantibodies may react to thyroid and neuronal antigens. Diagnosis is further complicated by the nonspecific neuroimaging and CSF results. Therefore, a high level of suspicion is required when a patient has thyroid autoimmunity and neuropsychiatric symptoms that cannot be explained by other means (37–40).

### 4.2 Key points for differential diagnosis from typical AE

The typical antibody or tumor association is often accompanied by typical AE (e.g., anti-NMDAR encephalitis with ovarian teratoma). However, evidence of the tumor is usually absent in patients with HE, and thyroid autoantibody positivity is a diagnostic clue for HE.

Making a distinction between HE and the types of AE is necessary as their pathology and management differ. Typical AE is defined as a group of syndromes with an immune basis, and specific diagnosis is made by demonstration of the antibodies to the neuronal surface antigens, such as anti-NMDAR antibodies. In addition, paraneoplastic associations, such as ovarian teratomas in young women, are common, and surgical removal of the tumor is part of the therapeutic approach, with better outcomes. On the other hand, HE, which is linked to HT, shows no tumor evidence, and its diagnosis is made on the basis of raised thyroid autoantibodies (TPOAb and TgAb), although these antibodies are not directly neuropathogenic. Differential diagnosis between HE and typical AE requires tumor screening for the latter, but not for the former, which is supported by retrospective data indicating that patients with AE are characterized by the presence of certain antibodies and tumors, while those with HE exhibit consistent positivity for thyroid autoantibodies without the presence of tumors (4, 41). Therefore, if encephalopathic patients have thyroid autoantibodies but no tumors or NSAbs, consideration should be given to HE so that the correct immunotherapy can be administered and unnecessary oncological interventions can be avoided. A critical issue in the field is whether HE/SREAT constitutes a truly independent clinical entity or represents an umbrella term for various autoimmune encephalopathies that happen to co-occur with thyroid autoantibodies. Unlike antibody-defined AE syndromes (e.g., anti-NMDAR, anti-LGI1, or anti-CASPR2 encephalitis), HE/SREAT lacks a specific pathogenic antibody, and its diagnosis relies on exclusion criteria and steroid responsiveness. The high prevalence of thyroid autoantibodies in the general population further complicates their use as diagnostic markers as they may reflect a general autoimmune predisposition rather than a direct neural pathogenicity. This diagnostic ambiguity has led some authors to question whether HE/SREAT should be reclassified once more specific biomarkers are identified.

The CSF profile in HE typically shows mild protein elevation and lymphocytic pleocytosis. However, oligoclonal bands (OCBs) are less frequently detected than in typical AE, and NSAbs are generally negative. CSF assessment is crucial for the diagnosis of both AE and HE. In addition, it offers a window into their immunopathology and is valuable for diagnosis. CSF changes reflecting CNS inflammation are common in AE, such as lymphocytic pleocytosis and elevated protein. Intrathecal antibody synthesis is evidenced by OCBs, which support a CNS immunoresponse and are quite common in AE. Diagnostic markers are provided by the neuronal antibodies (e.g., anti-NMDAR) through their presence in the CSF. The CSF picture in HE is milder with respect to protein and pleocytosis, and their increase is less than that found in AE. The same is then true for OCBs as their positivity rate is lower in HE, pointing to a different pattern of intrathecal immunity. Furthermore, the absence of neuronal antibodies, which is characteristic of HE, corroborates its different pathogenesis as the brain injury is due to thyroid autoimmunity rather than a direct antibody attack to neurons. This CSF pattern is indicative of a less vigorous immune process and is one of the reasons that make the diagnosis difficult. The literature points out that, although nonspecific inflammation can be present in the CSF of patients with HE, the lack of neuronal antibodies and the lower prevalence of OCBs still aid in the differential diagnosis of HE from AE; thus, diagnosis can be made by follow-up of encephalopathic patients who have thyroid autoimmunity (4, 42). Therefore, CSF examination along with antibody testing plays an important role in the distinction between HE and typical AE, which is necessary for an efficient diagnosis and treatment.

In cases of HE, electroencephalogram (EEG) usually reveals widespread slow waves or epileptiform discharges, whereas EEG in typical AE may show distinctive patterns such as delta brush. EEG is an extremely useful method for the examination of patients with encephalopathy, which can provide some understanding of brain behavior and facilitate the discrimination between HE and typical AE. In HE, EEG reveals primarily nonspecific, widespread slowing of signals, and features of epileptiform discharges may sometimes be seen. On the other hand, for typical AE, such as anti-NMDAR encephalitis, characteristic patterns including “extreme delta brush” exist, which can be used for early diagnosis. To explain the reason behind these EEG patterns in two groups of patients, one may say that it is the widespread brain dysfunction in HE against the selective synaptic disruption in AE that results in these phenomena (41, 42). Hence, EEG reading along with clinical and laboratory evidence is beneficial to discriminate HE from typical AE so that the right therapeutic approaches can be implemented (**Table 1**).

**Table 1:** Comparison of the key features between Hashimoto’s encephalopathy (HE)/steroid-responsive encephalopathy associated with autoimmune thyroiditis (SREAT) and classic autoimmune encephalitis (AE).

Feature	HE/SREAT	Classic AE
Defining antibody	Nonspecific (thyroid Abs present, but not pathogenic)	Neuronal surface antibodies (e.g., anti-NMDAR, anti-LGI1)
Pathogenic mechanism	Unclear; thyroid Abs may be markers	Direct antibody-mediated neural dysfunction
Paraneoplastic association	Generally absent	Common in certain subtypes (e.g., ovarian teratoma in anti-NMDAR)
CSF findings	Mild protein elevation; OCBs less frequent; NSAbs negative	Lymphocytic pleocytosis; OCBs common; NSAbs positive
MRI findings	Normal or nonspecific white matter changes	Medial temporal/limbic hyperintensities (in many subtypes)
EEG patterns	Nonspecific slowing; occasional epileptiform discharges	Characteristic patterns (e.g., “extreme delta brush” in anti-NMDAR)
First-line treatment	Corticosteroids	Corticosteroids + IVIG/plasma exchange; tumor removal if paraneoplastic

*CSF*, cerebrospinal fluid; *NMDAR*, *N*-methyl-d-aspartate receptor; *Abs*, antibodies; *OCBs*, oligoclonal bands; *NSAbs*, neuronal surface antibodies; *EEG*, electroencephalogram; *IVIG*, intravenous immunoglobulin.

### 4.3 Imaging features

The brain MRI of patients with HE sometimes reveals no abnormalities, but may also show nonspecific white matter hyperintensities, brain atrophy, or cortical edema. Unlike the typical medial temporal or limbic system involvement common in AE, these manifestations are variable and nonspecific. Brain MRI is the mainstay of evaluation in autoimmune encephalopathies, and HE is one of its manifestations, albeit a rare one, linked to HT. The feature of typical AE on MRI is often characteristic of the involvement of the medial temporal lobes or the limbic system, whereas normal images or nonspecific abnormalities are more the rule in HE. In a number of cases, the MRI findings in HE have been reported as normal, and where abnormalities were present, these consisted of diffuse or patchy white matter hyperintensities on T2-weighted or fluid-attenuated inversion recovery (FLAIR) sequences, which are only representative of nonspecific white matter changes rather than local limbic involvement (43). In order to depict the neurodegenerative or inflammatory process that is occurring, atrophy and edema are the two main changes that have been reported in a number of HE cases, which are more a widespread process rather than the limbic inflammation typical of AE (43). This is at variance with the traditional imaging pattern of AE, where hyperintensities are most often confined to the medial temporal lobes or the hippocampi or other limbic structures and are correlated with the clinical syndrome of limbic encephalitis (44). Because of the differences and the non-specificity of the MRI findings in HE, diagnosis relies on weightage of the clinical, serological, and imaging data altogether. More so, the lack of overt limbic lesions on MRI in patients with HE may cause the disease to be missed or not recognized, which means that HE should still be considered as a possibility in patients presenting with encephalopathy and nonspecific MRI changes who also have thyroid autoimmunity (9). The explanation for how the HE imaging pattern differs from other types of AE is likely to be found in the differences in the autoimmune targets and immune mechanisms. To summarize, brain MRI in HE is an adjunct diagnostic tool, providing support rather than being definitive; therefore, normal or nonspecific MRI findings do not rule out the diagnosis.

Functional imaging (e.g., PET-CT) can highlight areas of diminished metabolism or activity in the frontal lobes, temporal lobes, or cerebellum, thereby helping clinicians estimate the extent and severity of brain functional impairment.

Neurofunctional imaging modalities, in particular fluorodeoxyglucose positron emission tomography–computed tomography (FDG-PET/CT), are increasingly being used as supplementary diagnostic tools in AE and related disorders, including HE. FDG-PET/CT is a technique that enables imaging of the cerebral glucose metabolism and, hence, neuronal activity. It is possible that PET imaging could detect abnormalities even in those patients whose MRI scan remains unremarkable (45). This goes hand in hand with the phenomenon of brain dysfunction that may underlie such clinical features of HE, such as cognitive dysfunction, psychiatric manifestations, seizures, and ataxia (43). Therefore, the diagnostic sensitivity is increased when FDG-PET is considered together with MRI, the latter being normal or nonspecific. Moreover, metabolic PET abnormality can guide in monitoring the disease activity and the response to immunotherapy, as patients who clinically recover show not only improvement but also normalization of metabolic changes, which in turn indicates that brain inflammatory injury is reversible (46). Furthermore, PET-CT is a useful tool to rule out neoplastic causes in paraneoplastic AE, which may be HE or HE mimics (47). By integrating functional imaging data with clinical and laboratory information, we can improve our understanding of the pathophysiology and extent of brain function in HE and AE. This will allow developing more targeted strategies for treatment and more accurate methods of prognosis.

Repeated brain imaging studies of patients with HE have shown that some of the lesions on MRI can be destroyed by immunotherapy, indicating that there is room for changes in the inflammation process. With the help of serial imaging investigations in HE, vital information has been obtained about the evolving nature of brain lesions and the possibility that these inflammatory lesions can be reversed with timely and appropriate treatment. The initial MRI for HE may be normal or demonstrate nonspecific changes of white matter hyperintensities. However, after immunotherapy, repeat imaging frequently shows a marked improvement or the absence of these lesions, thus pointing to the plasticity of the underlying inflammatory process (43). This is what sets it apart from neurodegenerative or ischemic lesions, the hallmark of which is permanent structural damage. For example, in some patients with HE, MRI has shown diffuse subcortical lesions or limbic system changes, which have actually reduced or completely disappeared after corticosteroid or immunoglobulin therapy, thereby matching clinical recovery (43). Such results are very much in favor of the notion that HE is basically an AE, with inflammatory edema and demyelination being the main mechanisms, rather than an irreversible neuronal loss. Similarly, in other forms of AE, functional imaging studies have documented normalization of hypometabolic areas on FDG-PET concurrent with clinical improvement, further reinforcing the concept of immune-mediated brain dysfunction amenable to immunomodulation (46). There are numerous clinical and therapeutic consequences of imaging changes reversibility. These basically underline the importance of the early identification of the disease and the prompt initiation of immunotherapy in order to prevent permanent neurological complications. In addition, sequential imaging can be used as an objective biomarker to assess the effects of treatment, thus helping in making further therapeutic decisions. However, it is also likely that the extent of reversibility is dependent on various determinants, such as disease duration, severity, and comorbidity, among others. Essentially, dynamic imaging follow-up illustrates the therapeutic potential of recovery in HE and other autoimmune encephalopathies, which not only demonstrates the underpinning immunopathogenesis but also directs clinical management.

## 5 Diagnostic challenges of HT and AE

### 5.1 Differences and overlaps in diagnostic criteria

The diagnosis of HE and AE is challenging as these two conditions share considerable clinical and laboratory overlap, in particular in patients with underlying HT. In the main, the diagnosis of HE follows the guidelines by Graus et al., in which the following features are highlighted: encephalopathy or brain dysfunction, presence of thyroid autoantibodies, excluding the symptoms that are caused by other factors, and a favorable response to immunotherapy (43). However, as thyroid autoantibodies are extremely common in patients with HT, their diagnostic power is limited. As a result, a diagnosis of HE may be made in other encephalopathies that are not actually HE. Typical AE, on the other hand, mainly involves identifying neuronal surface autoantibodies, including anti-NMDAR antibodies (34, 48). The immunological overlap between HT and AE further complicates clinical differentiation—there is a group of patients with AE who do not show positive results to the known antibodies, while patients with HT may have increased neuronal autoantibodies without the classic symptoms of AE. Brain MRI changes are generally not highly specific in HE, while AE may present local abnormalities. EEG changes are frequent in both disorders, however are not specific to the disease (43, 49). To conclude, there is a large overlap in the criteria for the diagnosis of HE and AE when a patient has HT. Due to the presence of thyroid autoantibodies in HE, there is a decrease in specificity, whereas in AE, neuronal autoantibody detection comprises the main criteria, which may not be available in some cases. A thorough approach is highly beneficial in this situation, including clinical features, antibody tests, ensuring that no other conditions are responsible, and assessing how a patient’s symptoms change after immunotherapy. Furthermore, future research should be directed toward determining more specific biomarkers to differentiate HE from AE in patients with HT.

### 5.2 Limitations of laboratory testing

Laboratory testing is the cornerstone for the identification and management of HT and AE, including HE. However, there are a number of drawbacks that limit their effectiveness at accurately illustrating the disease progress or severity. One of the main issues is that both TPOAb and TgAb are increased in HT, but do not correspond to the severity of HE or other neuropsychiatric symptoms (43). Therefore, their utility as markers for disease activity or prognosis is significantly limited. CSF and serum investigation for NSAbs are absolutely necessary for the diagnosis of AE. However, commercially available indirect immunofluorescence assays (IIFAs) have shown significant false-negative rates: up to 29% of CSF samples with NSAbs could be missed, and this is especially true for the GABAB receptor, LGI1, and AMPA receptor antibodies (50). In the case of HE, NSAb test results tend to be negative, although occasionally, low-titer or new antibodies that cannot be detected by standard methods may be present. Molecules that indicate inflammation, such as IL-6 and chemokine CXCL13, are upregulated in both HT and AE. However, these have such a low level of disease specificity that they cannot be used for differential diagnosis or disease monitoring. Overall, testing for thyroid and neuronal antibodies, as well as for inflammatory markers, is necessary. However, these tests have some major deficiencies, which include: no association between the thyroid antibody titers and the HE severity; the NSAb assay results being often false-negative; and nonspecific inflammatory markers. All of these factors contribute to the less than perfect diagnostic and prognostic utility of these tests. These shortcomings compel comprehensive clinical judgment along with advanced laboratory methods to obtain higher diagnostic precision in HT linked with autoimmune encephalopathies.

### 5.3 Common causes of misdiagnosis and missed diagnosis

Due to its varied and nonspecific manifestations, HE, otherwise called SREAT, is a massive diagnostic issue. It is well documented that more than 50% of cases are initially misdiagnosed (11, 51, 52), which is mainly due to HE symptoms very closely mimicking those of viral encephalitis, primary psychiatric disorders, dementia, and metabolic encephalopathies. Patients with HE can show acute/subacute encephalopathy with cognitive dysfunction, behavioral changes, seizures, and neuropsychiatric symptoms including hallucinations, delusions, and psychosis. Sometimes, thyroid dysfunction in patients with HT, such as hypothyroidism or hyperthyroidism, makes the doctor think that the neurological symptoms are caused only by the thyroid hormone changes and not by AE. However, quite a few patients with HE have thyroid hormone levels within the normal range or have only slight abnormalities—in fact, the encephalopathy is mediated by autoimmunity and is not directly dependent on the thyroid hormone levels (1, 51). An important systemic factor responsible for misdiagnosis is the absence of a standard diagnostic protocol along with the lack of multidisciplinary work between endocrinologists, neurologists, psychiatrists, and immunologists. HE calls for a broad evaluation that should incorporate detailed clinical assessment, exclusion of alternative causes, measurement of anti-thyroid antibodies, CSF analysis, and neuroimaging (14, 51). As there are no agreed diagnostic criteria, patients often receive fragmented care and recognition is delayed. However, it has been proven that a multidisciplinary approach of working changes the situation for the better, leading to improved diagnosis and patient welfare (53). In summary, the major reasons behind the high misdiagnosis rates of HE are its clinical similarity to other encephalopathies, the puzzling effects of thyroid hormone abnormalities, and the lack of standardization of the diagnostic pathways. The measures for these challenges include heightened awareness, routine testing for anti-thyroid antibodies in unexplained neuropsychiatric cases, and the adoption of comprehensive diagnostic protocols. Early and correct diagnosis can make a big difference as the patient can respond well to corticosteroid or immunosuppressive medications (11, 14, 51). It is important to acknowledge that much of the literature on HE/SREAT consists of individual case reports and small case series, which inherently limits the strength of the evidence. Future research should prioritize multicenter cohort studies, systematic reviews, and the development of consensus diagnostic criteria to improve the quality of evidence in this field.

### 5.4 Proposed diagnostic pathway for autoimmune encephalitis in patients with Hashimoto’s thyroiditis

**Figure 2:**
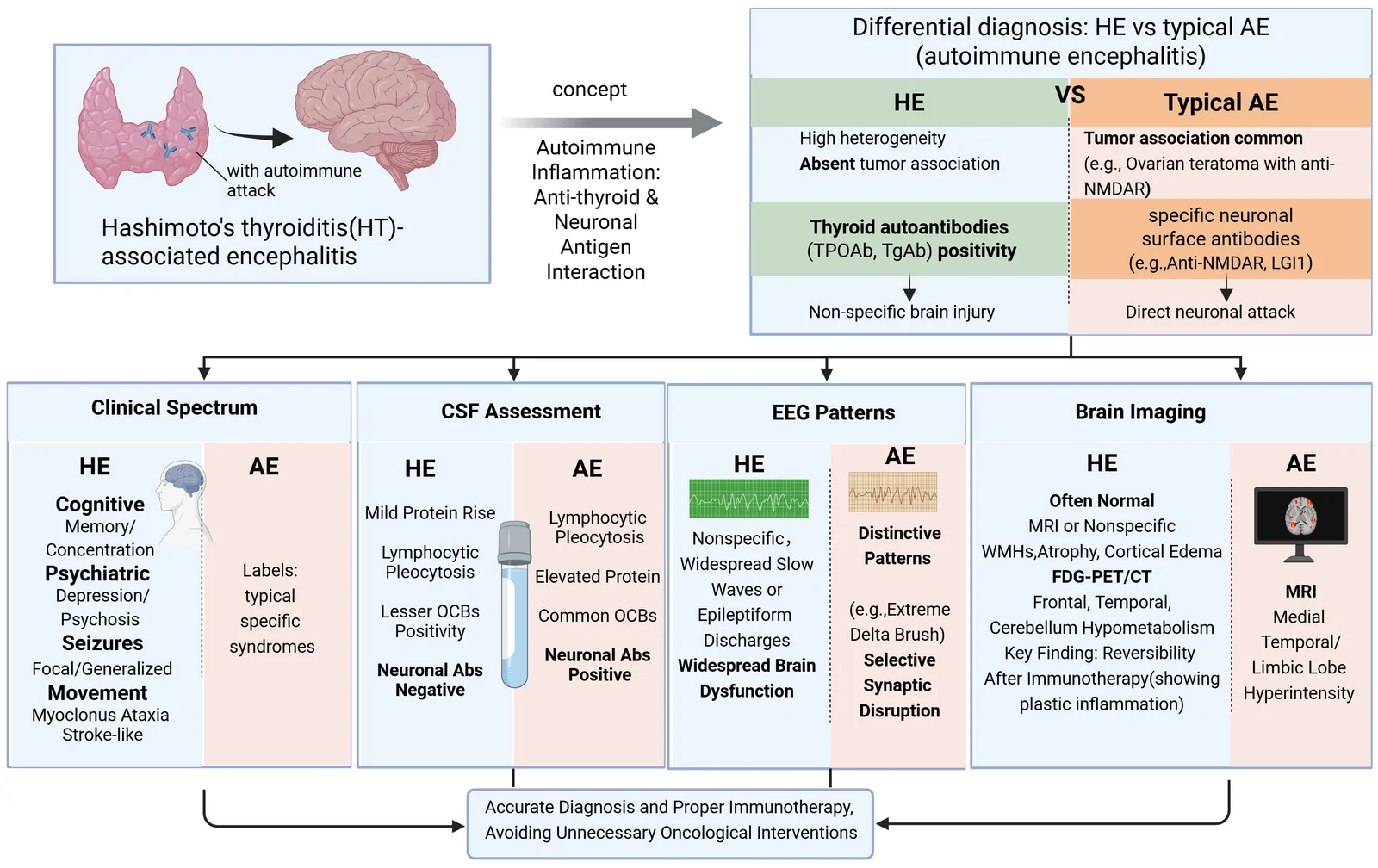
Clinical landscape and diagnostic distinctiveness of Hashimoto’s encephalopathy.

For patients with HT and suspected AE, we propose a structured yet streamlined diagnostic pathway. Initial evaluation comprises a joint endocrinologic and neurologic assessment, with attention to cognitive, psychiatric, and neurological signs, supplemented by a thyroid function test and a thyroid autoantibody panel. CSF analysis—including cell count, protein, glucose, OCBs, and NSAbs—together with gadolinium-enhanced brain MRI and EEG is essential to detect limbic or epileptiform patterns and to identify therapeutically relevant AE subtypes. Screening for paraneoplastic causes should be pursued when indicated before the diagnosis of SREAT is considered. Given the low specificity of elevated TPOAb titers and the inability of the current criteria to predict steroid responsiveness, no single test reliably distinguishes HE/SREAT from AE, and diagnosis must therefore be inherently iterative and multidisciplinary, involving an endocrinologist, neurologist, psychiatrist, and an immunologist or rheumatologist, with early oncologic consultation when paraneoplastic disease is suspected. After systematically excluding classical AE through NSAb and paraneoplastic screening, a trial of corticosteroid therapy in antibody-negative patients with compatible presentations—with reassessment based on treatment response—should guide the diagnosis. This approach thus underscores the systematic exclusion of classical AE before assigning HE/SREAT and promotes multidisciplinary collaboration from the outset rather than as a last resort (**Figure 2**).

## 6 Treatment strategies for HT and AE

### 6.1 Immunosuppressive therapy

Immunosuppressive therapy is the principal treatment for AE including HE, which is highly associated with HT. It is typically the first step in the management of patients, and high doses of corticosteroids, such as methylprednisolone pulse therapy, have been used as the first-line therapy (54). Early initiation of high-dose corticosteroid therapy is essential to optimize neurological outcomes. When glucocorticoids fail to control the disease or when the disease relapses, cyclophosphamide, mycophenolate mofetil, and rituximab are the second-line immunosuppressants used in HE and other forms of AE. Rituximab is a monoclonal anti-CD20 antibody that depletes B cells, which are thought to be the main contributors to the pathogenesis of antibody-mediated encephalitis (55). Adjunctive therapies such as IVIG and plasma exchange have been shown to be highly effective not only in HE cases but also in AE, particularly in severe ones or when corticosteroids are contraindicated (56). A number of case reports have recorded, upon treatment with IVIGs, seizure and cognitive improvement in patients with HE, even in those who were resistant to steroids (9). Novel drugs aimed at the immune system have been developed, including monoclonal antibodies against B cells, IL-6 (tocilizumab), and complement components (eculizumab) (57, 58). Nevertheless, further research is needed before these drugs can be made available for HE. Although various immunosuppressive agents are available, the best treatment regimen for HE has yet to be determined due to the disease being extremely rare. The majority of the existing data are derived from retrospective studies and case series, highlighting the need for well-designed prospective, controlled trials. Obtaining a diagnosis without delay and starting immunotherapy as soon as possible are key to neurological recovery.

### 6.2 Thyroid function management

Restoring normal thyroid function is the mainstay therapy for AE with HT, also known as HE. In patients with hypothyroidism, levothyroxine is administered in order to achieve euthyroid status, whereas those with hyperthyroidism are treated with anti-thyroid drugs to keep their thyroid hormone levels within the normal range. However, according to clinical data, simply getting the thyroid hormone levels back to normal will not completely resolve or alleviate the encephalopathic symptoms in HE, which means that the immune mechanisms that cause the disease are also effective independently of the action of thyroid hormones (9, 59). Assessment of thyroid autoantibodies can be done when there is a need to determine an immune profile and to decide on the appropriate immunotherapy. HT and HE are both characterized by high levels of antibodies, but in themselves are not very specific (1). A study demonstrated the prognostic value of a low free triiodothyronine (FT3) concentration, which is associated with a more severe neurological impairment and a worse outcome (60). In reality, the management of thyroid function in HE is essentially a matter of two things: one is to fix the hypothyroidism or hyperthyroidism to get the patient’s overall metabolism functioning well and the other is the use of immunomodulatory therapies such as corticosteroids, IVIGs, or other immunosuppressants to tackle the main issue of AE (1). In conclusion, thyroid function management in HE involves more than just normalization of the hormone levels. While levothyroxine supplementation or anti-thyroid drugs are indispensable for correcting thyroid dysfunction, neurological symptoms often remain, in which case immunotherapy targeting the autoimmune mechanisms is required. In addition, continuous monitoring of the thyroid autoantibodies, thyroid hormone levels, and the patient’s clinical status is of utmost importance to maximize treatment benefits.

### 6.3 Symptomatic treatment and rehabilitation

The management of AE associated with HT through symptomatic treatment and rehabilitation is essential to address seizures, psychiatric symptoms, and cognitive defects. Epileptic seizures are among the most frequent symptoms of AE. Anti-epileptic drugs (AEDs) such as levetiracetam are typically prescribed for seizure control. Nonetheless, in some individuals, the seizures may resolve post-immunotherapy treatment, thereby leading to the gradual discontinuation of AEDs (61, 62). Psychiatric symptoms including depression and anxiety often continue long after neurological stabilization. Selective serotonin reuptake inhibitors (SSRIs) are frequently used; however, physicians need to be very careful due to possible drug interactions with immunosuppressive agents such as corticosteroids and rituximab (63, 64). There should be collaborations between experts such as neurologists, psychiatrists, and psychologists. Cognitive dysfunction is one of the main features of AE, which affects memory, attention, and executive function. These cognitive rehabilitation programs have been shown to be highly effective, although some resistant defects have been observed (65, 66). A patient’s motor abilities can also benefit significantly from physical therapy. Emotional and psychological support goes along well with these modalities. In fact, an early start of rehabilitation is associated with the best functional recovery (65, 67). Collectively, treatment of the symptoms and rehabilitation of patients with AE should be comprehensive and involve a team of different specialists. Seizure control will still rely largely on AEDs; however, with the administration of immunotherapy, seizure remission could be possible. SSRIs have been proven helpful in the management of psychiatric symptoms. Consideration of the drug interaction issues is a must. Cognitive rehabilitation, physical therapy, and psychological support should all be viewed as integral components of post-encephalitic care and as a means of promoting long-term improvement.

## 7 Prognosis and follow-up of HT and AE

### 7.1 Prognostic factors influencing outcomes in HT-associated autoimmune encephalopathy

Treatment commencement and diagnosis at an early stage are the most important things to consider for HT-associated AE cases. Delays in treatment can result in irreparable damage to the nervous system and in cognitive deterioration, which is why doctors must be attentive to the symptoms and act swiftly (68). The age factor determines to a great extent the response to treatment and the risk of relapse. Moreover, older patients generally do worse from a prognostic point of view. This could be due to the fact that neuroplasticity is reduced in these patients, as are their body functions, and they typically suffer from other conditions such as diabetes and hypertension (69). Persistent elevation of thyroid autoantibody titers (TPOAb and TgAb) and altered CSF inflammatory markers are the main markers for prognostication. Long-term monitoring is warranted if high antibody titers are maintained as this strongly indicates relapse (68). Brain imaging is an additional method for determining prognosis. DTI has detected changes at the microscopic level to brain tissue in euthyroid HT individuals, which indicates possible subclinical CNS involvement (30). Overall, a number of factors such as the time of diagnosis and treatment, patient age, health status, and immune system markers are involved in determining the prognosis of HT-associated AE. The best way to achieve good clinical results comprises starting immunotherapy early and being very thorough, keeping comorbidities under control, and regularly checking the antibody levels and CSF markers.

### 7.2 Relapse and long-term management

HE, an extremely rare neurological side effect of HT, has a relapse rate ranging from approximately 20% to 30% based on the available case series and retrospective studies. Despite being the foundation of relapse prevention, long-term immunosuppressive treatment needs be regulated in order to not only achieve a balance between the desired effect of therapy and side effects but also to maintain the lowest effective dose of immunosuppressants that will preserve remission and at the same time limit toxicity risks (55, 70, 71). The detection of thyroid function abnormalities and autoantibody titers requires regular screening. MRI, as a neuroimaging technique, can be very handy in assessing neuroinflammation (72). Evidently, AE research has found that immunotherapy initiation and continuation are linked to favorable results, fewer relapses (higher probability of remission), and general improvement. Despite the entirely safe and generally effective treatment, the possibility of producing side effects exists. Therefore, different medications are utilized as alternative options, such as rituximab, which has been shown to result in a 14% relapse rate (55). To conclude, the therapeutic interventions in the case of a relapse of HE and the long-term approach have to be multidisciplinary with a keen awareness of the efficiency and the side effects of immunosuppressive therapy, complete with regular clinical and laboratory checks and neuroimaging to reveal signs of the disease early, thereby leading to a better patient prognosis and lower morbidity.

### 7.3 Quality of life and functional recovery

In the management of HE, quality of life (QoL) and functional recovery are the primary outcome variables. Immunotherapy, particularly corticosteroids and IVIGs, remains the cornerstone of treatment for this rare HT-associated neurological disorder. The majority of patients with HE show partial or complete improvement of cognitive function after appropriate immunotherapy, which consists of corticosteroids, IVIGs, or plasma exchange. Corticosteroids are essentially anti-inflammatory drugs. They are natural hormonal substances that help manage the body’s reaction to inflammation and regulate the immune response. IQ and memory testing starting from 6 to 12 weeks after the initial episode and then repeating every 6 months or so is recommended as the residual neuropsychological deficits following HE can be subtle and may be missed during a standard neurological examination. At present, pharmacological intervention is one of the main lines of treatment; however, it is recognized that social and psychological support networks are of fundamental importance in socialization and reintegration into patients’ lives. Neurologists, psychiatrists, neuropsychologists, occupational therapists, and social workers are members of the multidisciplinary team that provide the patient with a range of connected services to meet the patient’s needs (73, 74). Vocational rehabilitation involves occupational therapy and social therapy, which focuses on the reestablishment of desired or necessary roles and tasks in the community or workplace following injury or illness, with a special focus on work roles. Family education is a very important tool in supporting caregivers. Another major component of the treatment strategy is patient education, which is closely linked to improving the prognosis and reducing the relapse rate. Patients, as well as their families, can benefit from being well informed about their illness, taking their medications regularly, and practicing healthier lifestyles (75). In summary, Immunotherapy is the primary therapeutic method for remission in HE, but comprehensive care including cognitive rehabilitation, psychosocial support, and patient education is necessary to optimize QoL and functional recovery. The variety of clinical manifestations calls for personalized multidisciplinary approaches. Standardized outcome measures to quantify residual disability are needed going forward.

## 8 Future research directions of HT and AE

### 8.1 Discovery of novel biomarkers

Biomarker discovery for HE and AE is a priority goal in the field. Cutting-edge proteomic and metabolomic technologies have facilitated the processing of CSF and serum samples to identify hallmark biomolecules serving as diagnostic or prognostic tools. Metabolomic analysis of patients with HT has shown decreased levels of specific serum metabolites such as sphingomyelin (SM), lysophosphatidylcholine (LPC), and phosphatidylcholine (PC), which are involved in the pathogenesis of HT and may reflect systemic immune dysregulation that extends to CNS manifestations (76). Proteomic analyses have mainly focused on identifying novel autoantibodies and cytokine signatures. In fact, traditional thyroid autoantibodies (TPOAb and TgAb) do not specifically indicate CNS involvement (1). Therefore, researchers have continued their work in the direction of NSAbs and cytokine signatures. Two pro-inflammatory cytokines, i.e., IL-17 and IL-23, have been found at high levels in the serum of patients with HT, suggesting that these pro-inflammatory cytokines may lead to the immune augmented response in the CNS (77). Exploring cross-reactivity between thyroid autoantibodies and neuronal surface antigens is a very promising area. Certain thyroid autoantibodies may cause molecular mimicry or epitope spreading, eventually resulting in cross-reactive immune responses targeting neuronal proteins (4). More sensitive methods to detect very low amounts of antibodies are also being developed. At the same time, genetic markers of immune susceptibility (HLA haplotypes) have been on the radar of researchers for quite some time. Variations in the genes responsible for immune regulation, including *NLRC4*, are highly associated with susceptibility to HT and may also contribute to the risk of CNS autoimmunity (78). At the same time, the altered expression patterns of microRNAs (e.g., miR-146a, miR-142, and miR-301) in HT not only have been linked to the disease but also have been proposed as possible epigenetic markers (79). Taken together, the identification of new biomarkers for HE involves a holistic methodology combining proteomics, metabolomics, immunology, and genetics. While standard thyroid autoantibodies still serve as the main diagnostic tools, their inadequacy highlights the pressing need to determine much more specific markers. The development of high-throughput technologies provides new tools for enhancing early diagnosis and personalizing immunomodulatory treatments.

### 8.2 Establishment of animal models and mechanistic studies

The development of animal models reflecting the co-occurrence of HT and AE is a prerequisite for studying the common immunopathology of these diseases. Experimental autoimmune thyroiditis (EAT), brought about by thyroglobulin immunization, is able to reproduce HT-like lymphocytic infiltration and follicular destruction well (80). For modeling CNS involvement, this setup could be supplemented by CNS antigen co-exposure or adjuvants that enhance BBB permeability (80). Moreover, active immunization strategies against neuronal surface antigens have managed to induce encephalitis features, consisting of behavioral changes and the production of pathogenic antibodies (35). Combining these methods results in a dual pathology model representing a great tool to unravel the underlying neuro-thyroid autoimmune interplay. Gene knockout mouse models and other genetically engineered strains are especially useful to thoroughly investigate the molecular basis of HT-associated AE. IL-17 produced by Th17 is an important player: in models of AE after infection, Th17 cells trigger BBB breakdown and allow autoantibody entry, which causes neurological symptoms (22). Similarly, the loss or depletion of Tregs—through Foxp3 knockout or depletion—results in the breakdown of self-tolerance and therefore may induce cross-reactivity between thyroid and neuronal antigens (22, 81). These experimental approaches help prove molecular mimicry theories, where the same epitopes lead to joint systemic and central autoimmunity.

Adoptive transfer studies serve to dissect even more clearly how specific autoreactive T and B cells contribute to CNS pathology. The brains of animals have been shown to be infiltrated by B cells reactive to neuronal epitopes, which differentiate into antibody-producing plasmocytes and induce AE-like symptoms (35). On the other hand, when thyroid-specific T cells are transferred into mice, their capability to get through the BBB, attract inflammatory cells, and trigger cytokine cascades within the brain parenchyma can be studied (82). Alongside *in vivo* imaging, these cellular experiments identify particular lymphocyte subsets as potential therapeutic targets in HT-related neuroinflammation (82). Combined animal models and adoptive transfer studies offer a mechanism framework for how systemic thyroid dysfunction can disrupt central immune privilege. These systems highlight IL-17 signaling and Treg homeostasis as key intervention points for HT-related encephalitis.

### 8.3 Development of precision therapeutic strategies

The emergence of precision therapeutic strategies in the context of AE, including HE, considering the extent of an individual patient’s immune phenotypes, including the differentiation between Th17 cells and Tregs, and their specific autoantibody profiles is the main thrust of development of precision therapeutics. The rationale for individualized treatment lies in improving therapeutic efficacy while minimizing adverse effects. Immune phenotyping including evaluation of Th17/Treg balance is paramount as these two subsets serve totally opposite functions in autoimmunity: Th17 cells fuel inflammation, whereas Tregs are immune suppressors. An imbalance between these two subsets is involved in the pathogenesis of HT and AE (83). Antibody profiling plays an important role if a patient is to be treated individually. AE is marked by a variety of autoantibodies that target not only the neuronal surface but also intracellular antigens. Definitely, the presence and the levels of these antibodies are factors that affect the disease’s clinical characteristics, prognosis, and response to therapy (84). Targeted therapeutics are leading the way for future developments. Anti-IL-17 monoclonal antibodies have the potential to abolish Th17-driven inflammation. CTLA-4/immunoglobulin (Ig) fusion proteins interfere with T-cell co-stimulation, thus inhibiting the activation of autoreactive T cells. Bruton’s tyrosine kinase (BTK) inhibitors also prevent B-cell receptor signaling, which leads to a decrease in autoantibody production. Patients who do not get enough benefit from standard immunotherapies might be administered these drugs along with other medications (85). Among the new immunomodulatory therapies being tested are antigen-presenting tolerance and chimeric antigen receptor T-cell (CAR-T) therapy. Antigen-specific tolerance is designed to selectively dampen the activity of autoreactive lymphocytes while preserving overall immunity. CAR-T therapy is directed against autoreactive B cells or plasma cells that are responsible for producing harmful antibodies (85). To summarize, precision therapeutics for HE and AE entail: comprehensive immune and antibody characterization to guide patient-specific immunotherapy; targeted biologics such as anti-IL-17 monoclonal antibodies, CTLA-4/Ig fusion proteins, and BTK inhibitors; and discovery of novel therapies including antigen-specific tolerance induction and CAR-T therapy. The continuation of research and the initiation of clinical trials are imperative to establishing these treatment modalities. It is important to emphasize that CAR-T therapy, antigen-specific tolerance approaches, and BTK inhibitors remain largely experimental in the context of HE/SREAT. While these strategies hold promise for future precision immunotherapy, their clinical application in HE/SREAT patients awaits evidence from controlled clinical trials. Currently, the mainstay of treatment remains corticosteroids as the first-line therapy, with rituximab, IVIG, and plasma exchange as second-line or adjunctive options.

## 9 Conclusion

The association between HT and AE represents a compelling interface between endocrinology and neuroimmunology, illustrating the complexity of autoimmune diseases that transcend traditional organ boundaries. Based on currently available evidence, HE represents an intriguing example of the interface between thyroid autoimmunity and CNS inflammation. Based on epidemiological data, HE is a perfect example of thyroid autoimmunity and CNS inflammation, with four shared immunopathogenic mechanisms: molecular mimicry, T-cell-mediated immune dysregulation, BBB disruption, and NSAbs. These mechanisms highlight the systemic aspect of autoimmunity and the possible cross-reactivity of the thyroid and brain antigens. However, significant uncertainties remain regarding the pathogenesis of HE/SREAT, and the causal relationship between thyroid autoantibodies and neural injury has not been definitively established. Clinically, the diagnosis is still highly challenging as the patients‘ presentations greatly differ and pathognomonic features are missing. Therefore, the integration of endocrinology, neurology, and immunology is the key point of a multidisciplinary approach to the issue. Therapeutically, immunosuppression, mainly corticosteroids, is the basic pillar. Early treatment is linked to better results; however, relapse and long-term management remain major concerns. In summary, the case of HT and AE represents autoimmune diseases that can be manifested through different organs. One should be reminded that the proper and well-balanced approach, which is multidisciplinary integrating immunopathological knowledge with clinical expertise, is a must. Further scientific work, particularly on the discovery of specific biomarkers, the development of robust animal models, and well-designed clinical trials of precision therapeutics, will be essential to better understand and improve the clinical care of patients with this complex autoimmune overlap. Until then, the diagnosis and management of HE/SREAT should be approached with appropriate caution, recognizing the limitations of the current evidence base.
